# Characteristics, Assembly Processes and Stability of Bacterial Communities in Aquatic–Terrestrial Ecotone: A Case Study of Danjiangkou Reservoir, China

**DOI:** 10.3390/microorganisms14040923

**Published:** 2026-04-19

**Authors:** Xucong Lyu, Junjun Mei, Haiyan Chen, Huatao Yuan, Jing Dong, Xiaofei Gao, Jingxiao Zhang, Yunni Gao, Xuejun Li

**Affiliations:** 1 College of Fisheries, Henan Normal University, Xinxiang 453007, China; lvxvcong@outlook.com (X.L.); meijunjun2025@163.com (J.M.); happydj111@163.com (J.D.); xiaofeigao1989@163.com (X.G.); zhangjingxiao@htu.edu.cn (J.Z.); gaoyn@htu.cn (Y.G.); 2 Observation and Research Station on Water Ecosystem in Danjiangkou Reservoir of Henan Province, Nanyang 474450, China; haiyanch@126.com; 3 Ecological Environment Monitoring and Emergency Center of the Source of South-to-North Water Diversion Project in Henan Province, Nanyang 474475, China; 4 The National Ecological Quality Comprehensive Monitoring Station (Hebi Station), Hebi 458000, China

**Keywords:** aquatic–terrestrial ecotone, bacterial communities, community assembly, biogeochemical cycles, high-throughput sequencing

## Abstract

Aquatic–terrestrial ecotones are highly dynamic biogeochemical hotspots where hydrological fluctuations profoundly influence microbial community structure and ecosystem functioning. However, the mechanisms underlying microbial community responses across hydrological gradients remain insufficiently understood. In this study, 16S rRNA gene sequencing was used to comparatively analyze bacterial communities in the waterward and landward zones of the drawdown area of the Danjiangkou Reservoir. The results showed that bacterial community composition differed significantly between the two zones, and waterlogging markedly increased bacterial α-diversity. Community variation was primarily associated with key environmental factors, including total phosphorus (TP), soil moisture content (SMC), and nitrate nitrogen (NO_3_^−^-N). Compared with the landward zone, stochastic processes contributed more to community assembly in the waterward zone, which also exhibited higher network complexity and topological stability. In addition, several keystone taxa were identified, suggesting their potential roles in maintaining network structure and ecological stability. Functional prediction further revealed distinct metabolic potentials between zones, with enhanced anaerobic and redox-related functions in the waterward zone and predominantly aerobic metabolism in the landward zone. These findings suggest that hydrological fluctuations reshape bacterial community structure and potential ecological functions by jointly regulating water availability and nutrient dynamics. This study provides new insights into microbial ecological processes in reservoir riparian zones and offers a scientific basis for the management of aquatic–terrestrial ecotone ecosystems.

## 1. Introduction

The aquatic–terrestrial ecotone represents a critical transitional zone between aquatic and terrestrial ecosystems [1,2], playing a key role in maintaining ecosystem integrity and regulating biogeochemical processes such as material cycling, species migration, and energy transformation [3,4,5]. It also provides essential ecosystem services, including mitigating non-point source pollution, regulating water quality, and conserving biodiversity. In large reservoirs and lacustrine systems, water levels fluctuate substantially due to seasonal variation and anthropogenic regulation, leading to the formation of periodic drawdown zones characterized by alternating wet–dry cycles and frequent inundation–exposure events [6]. These hydrologically dynamic environments undergo continuous environmental reorganization, resulting in strong spatial heterogeneity in soil moisture, oxygen availability, and nutrient distribution [7,8]. Under the combined pressures of global climate change and human activities [9,10], water-level fluctuation (WLF) has emerged as a dominant driver shaping aquatic–terrestrial ecotones by regulating key ecological processes, including soil moisture dynamics, oxygen diffusion, nutrient cycling, and organic matter decomposition [11,12,13,14]. Despite its recognized importance, the mechanistic effects of WLF on microbial community organization and ecosystem functioning remain insufficiently understood, particularly in large, human-regulated reservoir systems.

Soil microorganisms, as the most active biological components of the aquatic–terrestrial ecotone [15], play a central role in driving the biogeochemical cycling of carbon, nitrogen, phosphorus, and sulfur [16,17,18]. Their metabolic activities not only regulate the efficiency and pathways of nutrient cycling but also exert profound impacts on water quality and greenhouse gas emissions [19]. Previous studies have demonstrated that microbial communities exhibit pronounced shifts in composition, functional redundancy, and metabolic preferences along moisture gradients [20]. In saturated environments, anaerobic processes such as denitrification, sulfate reduction, and methanogenesis are typically enhanced, whereas aerobic processes including organic matter decomposition, nitrification, and ammonia oxidation dominate under oxic conditions [21]. These functional shifts influence soil nutrient availability and have important implications for reservoir water quality and greenhouse gas fluxes [22,23,24]. However, despite these advances, a systematic understanding of how environmental drivers jointly regulate microbial functional potential, community assembly mechanisms, and ecological stability remains limited. Environmental fluctuations may disrupt microbial community structure by altering niche differentiation and the distribution of functional groups [25], while the expression of ecosystem functions depends not only on taxonomic composition but also on assembly processes and interspecific interactions [26]. Therefore, hydrological gradients driven by water-level fluctuations are likely to simultaneously influence microbial diversity, assembly mechanisms, and ecological interactions, yet their integrated effects remain insufficiently resolved.

Current ecological theories suggest that microbial community assembly is driven by a combination of stochastic processes and deterministic processes [27]. The relative balance of these processes reflects the potential dynamics of ecosystem stability and functional redundancy. Under intensified environmental stress, community assembly may shift from a deterministic mode dominated by environmental filtering to a stochastic mode driven by ecological drift [28,29]. Such shifts are often associated with declines in community stability and reduced predictability of ecosystem functions. However, clear evidence is still lacking regarding whether microbial assembly mechanisms undergo systematic restructuring under different hydrological conditions, particularly in drawdown zones—typical hydrologically dynamic environments. Furthermore, microbial communities are structured through complex metabolic coupling and cooperative/competitive interactions, forming intricate ecological co-occurrence networks [30]. The topological properties of these networks—such as modularity, connectivity, and the presence of keystone taxa—reflect structural stability and significantly influence functional maintenance [31,32]. Thus, a comprehensive analysis of the relationship between co-occurrence networks and potential metabolic functions is crucial for a holistic understanding of ecosystem regulatory mechanisms.

This study was conducted at the Danjiangkou Reservoir, China, one of Asia’s largest artificial freshwater reservoirs and the primary water source for the Middle Route of the South-to-North Water Diversion Project. The reservoir experiences pronounced seasonal water-level fluctuations and anthropogenic regulation, resulting in extensive riparian drawdown zones that alternate between inundation and exposure. These hydrologically dynamic areas provide an ideal natural laboratory for examining how hydrological gradients influence microbial ecological processes. In this study, we sampled two representative habitats—waterward (periodically saturated) and landward (relatively dry) zones—to explore the ecological processes and functional shifts of soil microbial communities under varying inundation stresses. Specifically, this study tested the hypotheses that (1) frequent inundation increases hydrological connectivity and microbial dispersal processes, thereby influencing bacterial diversity and the balance between stochastic and deterministic assembly processes; (2) hydrological heterogeneity restructures microbial interactions, leading to differences in co-occurrence network complexity and stability between habitats; and (3) environmental conditions associated with inundation drive differences in microbial functional potential across the hydrological gradient. This study provides new insights into how hydrological disturbances regulate microbial ecological processes in reservoir riparian zones. The findings contribute to a mechanistic understanding of microbial responses to water-level fluctuations and provide a scientific basis for ecosystem management and water quality conservation in large reservoir systems.

## 2. Materials and Methods

### 2.1. Study Area

The Danjiangkou Reservoir, the core water source for the Middle Route of China’s South-to-North Water Diversion Project, is one of the largest artificial freshwater lakes in Asia [33], which is located in the upper to middle reaches of the Han River, the reservoir spans Danjiangkou City in Hubei Province and Xichuan County in Henan Province. It has a total storage capacity of 29 billion cubic meters and a water surface area of approximately 1022 km^2^. Influenced by the monsoonal climate, the region receives an average annual precipitation of 800–1000 mm, with the majority falling between June and September. To meet water diversion demands, artificial regulation of the reservoir further intensifies water level dynamics, resulting in the formation of periodically exposed aquatic–terrestrial ecotones [34]. These ecotones exhibit a vertical elevation range of 10–30 m and can extend horizontally for several hundred meters.

**Figure 1:**
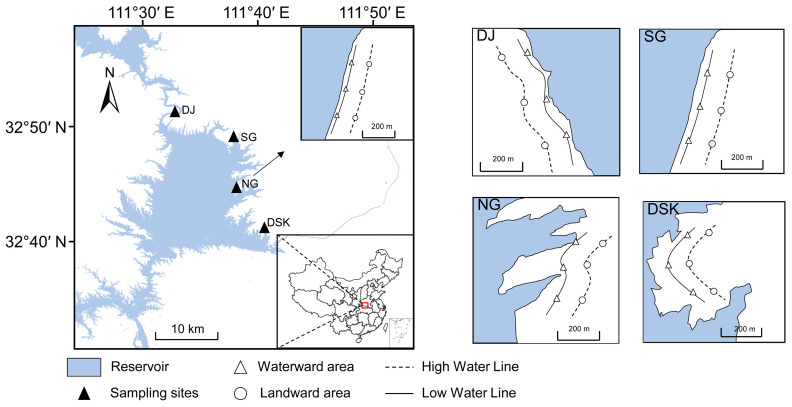
Sampling sites in the Danjiangkou Reservoir.

In this study, four primary regions of the Danjiangkou Reservoir were selected as sampling sites: Danjiang (DJ), the diversion reservoir (DSK), Nangan (NG), and Songgang (SG) (Figure 1 and Table S1). These regions were chosen to capture spatial heterogeneity across the reservoir and to ensure that the sampling design covered different hydrological and environmental conditions. To investigate the effects of water level fluctuations on bacterial community composition and functional characteristics within the aquatic–terrestrial ecotone, sampling sites were established at the high water line (frequently experiencing wet–dry alternation, defined as the landward zone) and the low water line (permanently inundated area, defined as the waterward zone).

### 2.2. Sample Collection and Soil Physicochemical Properties Analysis

Sampling was conducted in April 2024, when the reservoir was at its lowest water level, allowing the aquatic–terrestrial ecotone to be fully exposed. The distance between the high water mark and the low water mark was approximately 150 m. A stratified sampling design was applied across four regions and two habitat types (landward and waterward zones), resulting in a total of 24 samples (4 regions × 2 zones × 3 replicates). This design ensured both spatial replication and comparability between hydrological conditions. Surface soil samples were collected using the five-point composite sampling method. Although the total sample size was relatively limited, the inclusion of multiple regions and replicated sampling across contrasting hydrological zones ensured adequate spatial representation and comparability. Samples intended for soil DNA extraction were stored at −80 °C to preserve DNA integrity, while the remaining samples were stored at −20 °C for physicochemical analyses.

NO_3_^−^-N and NH_4_^+^-N were determined using a continuous flow analyzer (SEAL AA3). Total phosphorus (TP) and total nitrogen (TN) were measured using colorimetry and the Kjeldahl method after acid digestion. Calcium (Ca), magnesium (Mg^2+^), and iron (Fe) were analyzed using inductively coupled plasma optical emission spectrometry (ICP-OES). Soil organic matter (SOM) was determined using the potassium dichromate oxidation method. Soil moisture content (SMC) was measured by oven drying at a constant temperature of 105 °C.

### 2.3. DNA Extraction and Illumina Sequencing

Total DNA was extracted from soil samples using the DNeasy PowerSoil Pro Kit (Qiagen, CA, USA). The V3–V4 region of the bacterial 16S rRNA gene was amplified using the universal prokaryotic primers 341F (5′-CCTAYGGGRBGCASCAG-3′) and 806R (5′-GGACTACNNGGGTATCTAAT-3′) [35]. Gene library construction followed the method described by Liu et al. [36]. PCR products from all samples were pooled in equimolar amounts and, after quality control, sequenced by Novogene Co., Ltd. on the Ion S5TM XL platform (Thermo Fisher Scientific, Waltham, MA, USA). Unique barcodes were used to assign paired-end reads to individual samples, followed by preprocessing to remove barcode and primer sequences. Overlapping paired-end reads were merged using FLASH (v.1.2.11) [37] software to generate raw tag sequences. Quality filtering was performed using fastp (v.0.23.1) software to obtain high-quality clean tag sequences. Subsequently, tag sequences were aligned against the Silva database (v.138.2) using Vsearch (v.2.16.0) software to identify and remove chimeric sequences, yielding final effective tag sequences [38,39]. To ensure consistent sampling depth across microbial communities, all samples were subjected to rarefaction standardization. All the raw data from this study was deposited in the National Center for Biotechnology Information (NCBI) Sequence Read Archive (SRA) under BioProject ID PRJNA1333656.

### 2.4. Bioinformatic and Statistical Analysis

All data analysis and visualization were performed using R software (v.4.3.3). Alpha diversity indices, including Chao1, ACE, and Shannon, were calculated using the vegan package (v.2.6-2) [40], with significant differences assessed via Wilcoxon rank-sum test (*p* < 0.05). Beta diversity analysis was conducted based on the Bray–Curtis distance matrix, employing principal coordinates analysis (PCoA) with the vegan package to evaluate structural differences in bacterial communities between waterward and landward zones. Significance of community structure differences was tested using Permutational Multivariate Analysis of Variance (PERMANOVA) with 999 permutations (*p* < 0.05). Rarefaction curves were generated using the rarecurve function to confirm sufficient sequencing depth for capturing community diversity.

Distance-based redundancy analysis (dbRDA) was performed using the vegan package, with significant environmental variables selected via forward selection. The glmm.hp package (v.0.1-8) was used for hierarchical partitioning to quantify the contribution of each environmental variable to community structure [41]. Partial least squares path modeling was conducted using the plspm package (v.0.5.1) [42]. Bootstrapping (5000 iterations) was conducted to evaluate the stability and significance of the path coefficients.

Microbial co-occurrence network analysis was performed using the WGCNA package (v.1.73) [43]. Prior to network construction, the ASV matrix was rigorously filtered: only ASVs with relative abundance > 0.01% and present in at least 50% of samples were retained to minimize noise from rare taxa. Pairwise Spearman’s rank correlations were calculated, and network edges were retained using a threshold of |r| > 0.6 with Benjamini-Hochberg adjusted *p* < 0.01. A soft-thresholding power was selected to emphasize strong correlations, and network visualization was conducted in Gephi (v.0.9.1) using the Fruchterman-Reingold layout [44]. WGCNA was selected because it enables the identification of network modules and topological roles (e.g., module hubs and connectors), while providing a robust framework for comparing network complexity and stability across habitats. Keystone taxa were identified based on within-module connectivity (Zi) and among-module connectivity (Pi): nodes with Zi > 2.5 and Pi > 0.62 were classified as network hubs, nodes with Zi > 2.5 and Pi ≤ 0.62 as module hubs, and nodes with Zi ≤ 2.5 and Pi > 0.62 as connectors; all of these were considered keystone taxa.

To investigate community assembly mechanisms, the iCAMP package was used to quantify the relative contributions of ecological processes [45]. Beta Nearest Taxon Index (βNTI) and Raup-Crick Index (RCBray) were calculated based on a null model framework. |βNTI| > 1.96 indicated deterministic assembly (βNTI < −1.96 for homogeneous selection, βNTI > 1.96 for heterogeneous selection), while |βNTI| < 1.96 indicated stochastic processes. Within stochastic processes, RCBray < −0.95 indicated homogeneous dispersal, RCBray > 0.95 indicated dispersal limitation, and |RCBray| < 0.95 indicated drift (including weak selection, weak dispersal, diversification, and ecological drift).

## 3. Results

### 3.1. Differences in Bacterial Community Composition and Diversity Between the Waterward and Landward Zones

**Figure 2:**
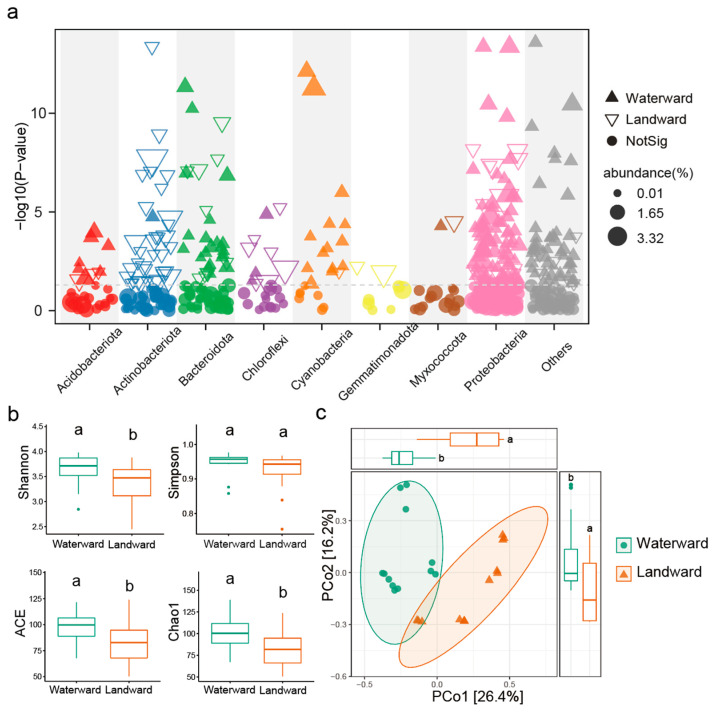
Shifts in the diversity and composition of the bacteriome between the Waterward and Landward zones. (**a**) Manhattan plots showed changes in the bacterial community in the Waterward and Landward zones at the phylum level. Gray dotted lines indicate the significance threshold (*p* = 0.05). (**b**) Box plot showed the α-diversity indices of the waterward and landward zones. (**c**) PCoA analysis based on Bray–Curtis distance. Different letters indicate significant differences.

High-throughput amplicon sequencing was performed, and after quality control, a total of 1,972,340 reads were obtained for all samples. A total of 40,876 ASVs were identified. The rarefaction curve indicated that the sequencing effort was sufficient to capture the majority of the bacterial diversity in the samples (Figure S1). Clear turnovers existed in taxonomic composition between the waterward zone and the landward zone (Figure 2a and Figure S2). The relative abundance at the phylum level showed that Proteobacteria was significantly more abundant in the waterward zone compared to the landward zone, while Actinobacteriota was significantly more abundant in the landward zone than in the waterward zone (Figure S3).

In terms of α-diversity, the Chao1 and ACE indices were significantly higher in the waterward zone than in the landward zone. The Shannon index was also significantly higher in the waterward zone, whereas no significant difference was observed for the Simpson index (Figure 2b). These results indicate that bacterial diversity was higher under waterlogged conditions. Principal Coordinates Analysis (PCoA) based on the Bray–Curtis distance matrix showed clear separation between the bacterial communities of the waterward and landward zones, suggesting significant communities structure differences between the two zones (Figure 2c, *p* < 0.05).

### 3.2. Drivers of Bacterial Community Dissimilarities Between the Waterward and Landward Zones

**Figure 3:**
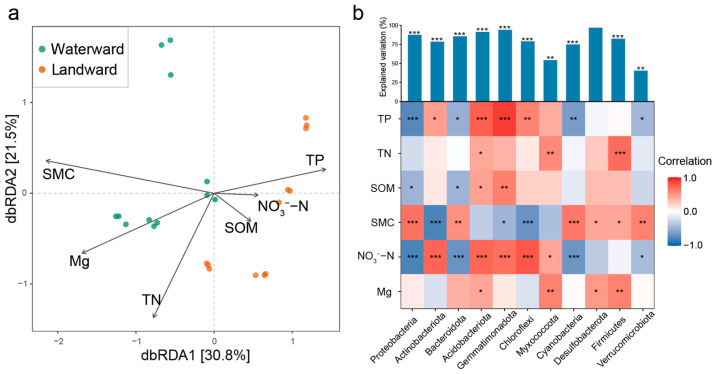
Driving force of soil physicochemical factors on bacterial communities. (**a**) dbRDA analysis of bacterial communities and environmental factors. (**b**) Correlation analysis between environmental factors and bacterial taxa. * *p* < 0.05, ** *p* < 0.01, *** *p* < 0.001.

The result based on distance redundancy analysis (dbRDA) showed that the microbial community structure in the aquatic–terrestrial ecotone was significantly driven by soil physicochemical factors (Figure 3a). The dbRDA1 axis explained 30.8% of the community variation, while the dbRDA2 axis explained 21.5%, suggesting that the combination of environmental variables can effectively account for the variation in bacterial community structure. Through forward selection, the key environmental factors influencing the bacterial microbiota in the aquatic–terrestrial ecotone soil were identified as total phosphorus (TP), total nitrogen (TN), magnesium (Mg^2+^), soil moisture content (SMC), soil organic matter (SOM), and nitrate (NO_3_^−^-N). Using Hierarchical partitioning analysis to further quantify the impact of environmental factors on bacterial communities, the results showed that NH_4_^+^-N, NO_3_^−^-N, TN, and Mg^2+^ were the main environmental factors in the Waterward zones, while Ca, Mg^2+^, TN, and TP were the main environmental factors in the Landward area (Figure S4).

Heatmap analysis further elucidated the microbial community’s response strategies to specific environmental factors (Figure 3b). Specifically, TP and NO_3_^−^-N were significantly positively correlated with Proteobacteria and Acidobateriota, but negatively with Gemmatimonadota and Actinobacteriota (*p* < 0.01). Similarly, SMC exhibited positive correlations with Proteobacteria and Cyanobacteria (*p* < 0.01) and negative correlations with Actinobacteriota and Gemmatimonadota (*p* < 0.05).

### 3.3. Waterlogging Reduces Deterministic Processes for Microbial Community Assembly

To evaluate the environmental adaptability and resource utilization breadth of bacterial communities, the niche breadth index was calculated using Levins’ formula. Bacterial communities in the waterward zone exhibited significantly broader niche breadths than those in the landward zone (Figure S5), suggesting that taxa in waterward habitats possess a higher capacity to utilize diverse resources or tolerate a broader range of environmental conditions.

**Figure 4:**
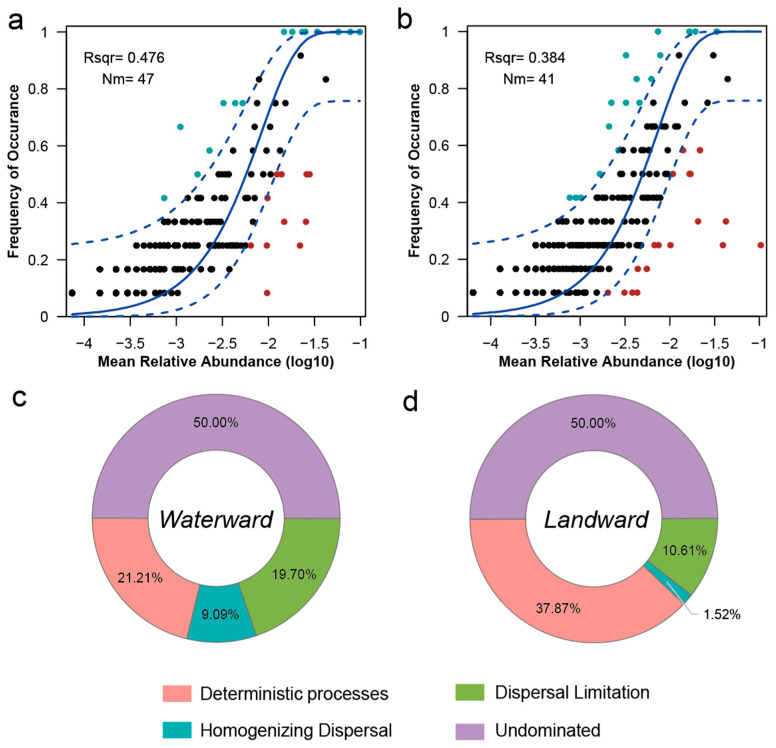
Analysis of microbial community assembly process. (**a**,**b**) show the neutral community model fitting curves for Waterward and Landward zones; (**c**,**d**) show the relative contributions of various ecological processes in the Waterward and Landward zones.

Neutral model analysis further suggested a stronger contribution of stochastic processes in the waterward zone, as indicated by a higher model fit (R^2^ = 0.476) compared with the landward zone (R^2^ = 0.384) (Figure 4a,b). In addition, the estimated migration rate was higher in the waterward zone (Nm = 47) than in the landward zone (Nm = 41), suggesting enhanced dispersal under inundated conditions. However, these moderate R^2^ values indicate that neutral processes alone cannot fully explain community variation and therefore do not support firm conclusions based solely on the neutral model; thus, we corroborated these findings using null model analysis, which provided consistent and quantitative confirmation. Stochastic processes dominated community assembly in both habitats; however, their relative contribution was higher in the waterward zone, where ecological drift accounted for 50.00% of the variation, followed by dispersal limitation (19.07%) and homogenizing dispersal (9.09%). In contrast, the contribution of deterministic processes increased to 37.87% in the landward zone, while homogenizing dispersal and dispersal limitation decreased to 1.52% and 10.61%, respectively (Figure 4c,d). These results indicate that while stochasticity prevails in both habitats, deterministic selection exerts a significantly stronger influence in the landward zone, whereas waterlogging (waterward) enhances the relative importance of stochastic assembly.

### 3.4. Waterlogging Enhances Microbial Network Stability and Complexity

**Figure 5:**
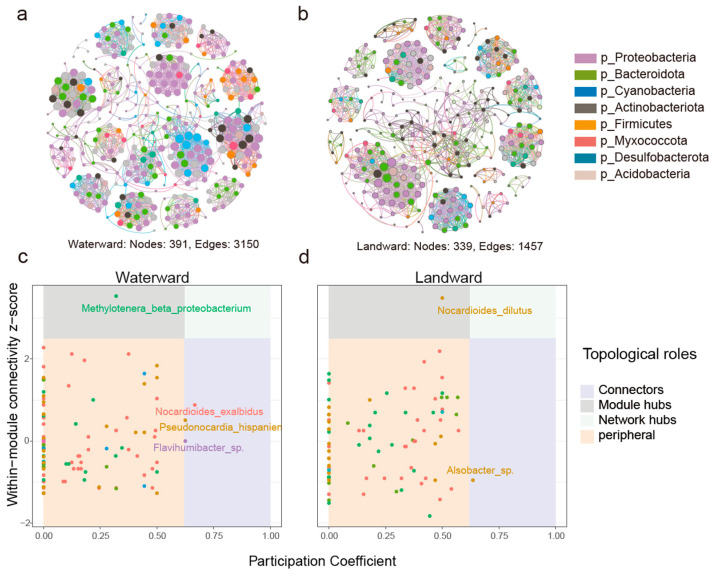
The complexity and stability of bacterial communities network between the Waterward and Landward zones. (**a**,**b**) Show the microbial co-occurrence networks in the Waterward and Landward zones, respectively; (**c**,**d**) Display the topological role distribution of bacterial species within the networks of the Waterward and Landward zones, respectively. Different colors represent different modules.

To characterize and assess the symbiotic interactions within the bacterial communities of the Waterward and Landward zones, a co-occurrence network was constructed, and the network’s topological properties were calculated. The co-occurrence network in the Waterward zone consisted of 391 nodes and 3150 edges, while the Landward zone network comprised 339 nodes and 1457 edges (Figure 5a,b). Robustness and stability analyses indicated that the Waterward network possessed superior resilience; it maintained higher connectivity during species or link removal, whereas the stability of the Landward network declined more rapidly (Figures S6 and S7).

Zi-Pi analysis revealed that in both the Waterward and Landward zones, most nodes were peripheral, with a few nodes acting as Module hubs and Connectors in each zone. No nodes functioned as Network hubs. Species identified as *Module hubs* and Connectors were identified as keystone taxa within the network (Figure 5c,d). In the Waterward zone, *Methylotenera_beta_proteobacterium* served as the *Module hub*, while *Nocardioides_exalbidus*, *Pseudonocardia_hispaniensis*, and *Flavihumibacter*_sp. were identified as Connectors. In the Landward zone, Nocardioides_dilutus was the Module hub, and *Alsobacter*_sp. was the Connector. These keystone taxa likely play critical roles in maintaining network stability and mediating ecological functions under varying hydrological conditions.

### 3.5. The Differentiation of Functional Profiles of Bacterial Communities Between the Waterward and Landward Zones

**Figure 6:**
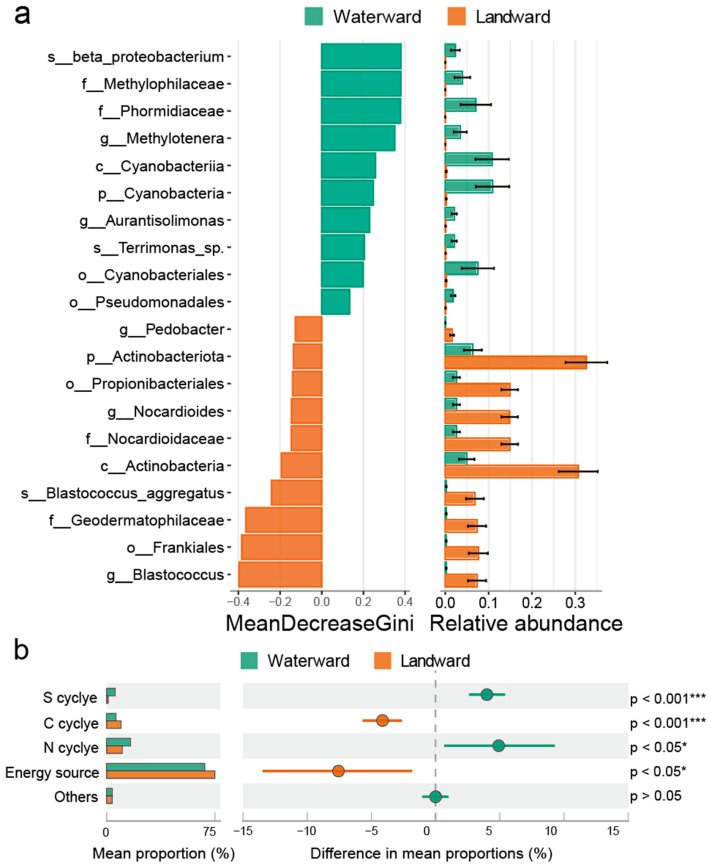
Functional Profiles of Bacterial Communities between the Waterward and Landward zones. (**a**) Indicator taxa identified by Random Forest analysis, highlighting taxa enriched in the waterward and landward zones. (**b**) STAMP analysis displaying functional differences between the Waterward and Landward zones.

To further elucidate the compositional and functional differentiation of bacterial communities between the waterward and landward zones, we employed a Random Forest analysis to identify zone-specific indicator taxa (Figure 6a). In the waterward zone, representative taxa were primarily affiliated with β-Proteobacteria (e.g., *s__beta_proteobacterium*, *f__Methylophilaceae*), methylotrophic genera such as *g__Methylotenera*, Cyanobacteria (e.g., *c__Cyanobacteriia*, *p__Cyanobacteria*), as well as genera including *Aurantisolimonas* and *Terrimonas*. In contrast, the landward zone was enriched with taxa from the phylum *Actinobacteriota*, such as *Nocardioides*, *Blastococcus*, *Geodermatophilaceae*, and members of the orders *Propionibacteriales* and *Frankiales*.

Consistent with these taxonomic differences, predicted functional profiles also differed between the two zones (Figure 6b and Figure S8). Functional prediction based on the FAPROTAX database indicated variations in microbial metabolic potential related to energy metabolism and biogeochemical cycling of carbon (C), nitrogen (N), and sulfur (S). Both zones showed relatively high proportions of functional groups associated with energy metabolism. However, the waterward zone exhibited higher relative abundances of functional groups associated with photoautotrophy and chemoautotrophy. Differences were also observed in predicted carbon cycling functions. Functional groups related to methane metabolism and aromatic compound degradation showed higher relative abundances in the waterward zone, whereas functional groups associated with cellulose degradation and methylotrophic metabolism were relatively more abundant in the landward zone. For nitrogen cycling, functional groups associated with nitrate reduction, denitrification, and nitrogen fixation showed higher relative abundances in the waterward zone, while functional groups related to ammonia oxidation were more abundant in the landward zone. In terms of sulfur cycling, the waterward zone exhibited higher predicted potential for sulfur respiration and sulfur oxidation, whereas the landward zone showed slightly higher relative abundance of sulfate respiration-related functions.

### 3.6. The Effects of Biotic and Abiotic Factors on the Community Stability

**Figure 7:**
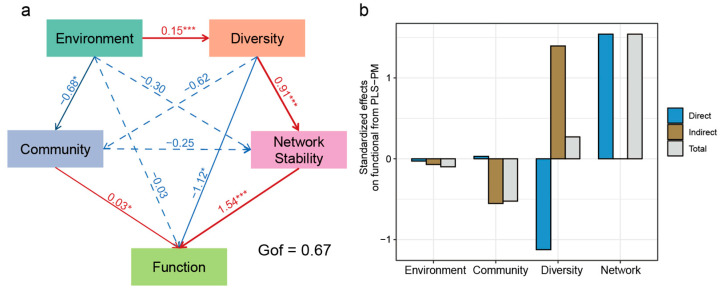
Contribution of biotic and abiotic factors to microbial community stability. (**a**) Path analysis based on environmental factors, diversity, community, stability, and function, with red lines indicating positive correlations, blue lines indicating negative correlations, and dashed lines representing non-significant relationships (* *p* < 0.05, *** *p* < 0.001); (**b**) Direct, indirect, and total effects of the PLS-SEM analysis.

To holistically integrate the complex interrelationships among environmental variables (Environment), bacterial community composition (Community), α-diversity (Diversity), network stability (Network), and potential metabolic functions (Function), a path analysis model was constructed. The model exhibited a high goodness-of-fit (GOF = 0.67), and the bootstrap results confirmed the stability of the model estimates. The results revealed that environmental factors exerted a significant positive effect on α-diversity but showed strong negative impacts on community composition and network stability. Notably, the direct effect of Environment on Function was weak and statistically insignificant (Figure 7).

Community composition was found to negatively influence both α-diversity and network stability, while exhibiting a significant positive effect on Function. Simultaneously, α-diversity showed a significant positive correlation with network stability, suggesting that higher taxonomic richness bolsters the structural robustness of microbial networks. Network stability, in turn, significantly and positively drove Function, implying that stable interspecies interactions are fundamental to maintaining microbial metabolic potential. Beyond direct pathways, Environment indirectly modulated Function through a cascade involving shifts in community composition, α-diversity, and network stability.

## 4. Discussion

### 4.1. Water Level Fluctuations Induced Different Soil Environments and Microbial Communities

The aquatic–terrestrial ecotone is a highly dynamic ecosystem in which periodic flooding and drying cycles generate strong spatial heterogeneity in environmental conditions. In this study, key environmental variables—including soil moisture, organic matter, and nutrient availability—differed significantly between the waterward and landward zones, thereby shaping the distribution and diversity of microbial communities (Figure 3). Such environmental gradients are widely recognized as major drivers of microbial community differentiation in reservoir riparian zones and other fluctuating hydrological systems [46]. Across hydrologically dynamic ecosystems, including riparian soils and floodplain wetlands, moisture gradients and redox fluctuations have consistently been identified as primary determinants of microbial spatial organization, underscoring the general ecological relevance of the patterns observed here [20,47].

Our results showed that the waterward zone, which experiences prolonged inundation and relatively higher nutrient availability, exhibited higher bacterial α-diversity and was dominated by Proteobacteria and Bacteroidota (Figure 2). These taxa are commonly associated with nutrient-rich and dynamic environments due to their metabolic versatility and rapid growth strategies [48]. In contrast, the landward zone, characterized by lower soil moisture and more stable terrestrial conditions, was enriched in Actinobacteriota and Gemmatimonadota, groups often reported to dominate in relatively dry soils and environments with more complex organic substrates [49]. Proteobacteria are widely recognized as copiotrophic bacteria capable of rapidly exploiting available nutrients. Their higher abundance in the waterward zone is likely associated with elevated concentrations of TP and NO_3_^−^-N, which may enhance resource availability and promote the proliferation of fast-growing taxa [50]. By contrast, Actinobacteriota are well known for their ability to degrade complex organic compounds through extracellular enzyme production, enabling them to thrive in comparatively stable and aerated soils [51,52]. Correlation analysis further supported these ecological patterns. TP and NO_3_^−^-N were positively correlated with Actinobacteriota and Gemmatimonadota, but negatively correlated with Proteobacteria and Acidobacteriota, indicating that nutrient effects are mediated through environmental context rather than acting as simple drivers of copiotrophic taxa dominance [53]. This divergence between taxonomic dominance and correlation patterns suggests that microbial responses to hydrological gradients involve coupled effects of resource availability and redox constraints, a phenomenon also noted in studies of heterogeneous soil systems [54].

Similar patterns have been reported in other reservoir systems. For example, studies in the Three Gorges Reservoir have documented the dominance of Proteobacteria in periodically flooded soils, which has been attributed to high nutrient inputs and fluctuating redox conditions [55]. However, compared with the stronger hydrological disturbances observed in that system, the Danjiangkou Reservoir experiences relatively moderate and seasonally regulated water level fluctuations. This may produce a more balanced microbial response across the ecotone. Together, these findings highlight that both the intensity and frequency of hydrological disturbance play critical roles in shaping microbial community composition in reservoir riparian ecosystems.

### 4.2. The Assembly Mechanisms of Bacterial Communities

Microbial community assembly in the aquatic–terrestrial ecotone is governed by the interplay between stochastic and deterministic ecological processes [45]. Our results showed that stochastic processes dominated community assembly in both habitats, with ecological drift accounting for approximately half of the observed variation. However, deterministic processes contributed substantially more in the landward zone than in the waterward zone (Figure 4). This pattern can be explained by differences in environmental stability along the hydrological gradient. In the waterward zone, frequent flooding and water level fluctuations create a highly dynamic environment in which microbial communities are repeatedly disturbed. Such disturbances can weaken environmental filtering and enhance the influence of stochastic processes, including ecological drift and dispersal [56,57]. As a result, community composition becomes less predictable but may exhibit greater flexibility under fluctuating environmental conditions. In contrast, the landward zone experiences relatively stable terrestrial conditions, which strengthen environmental filtering and promote niche differentiation among microbial taxa [58]. Under these circumstances, deterministic processes such as environmental selection play a larger role in structuring microbial communities. This shift along the stochastic–deterministic continuum aligns with observations from river–floodplain systems, where increased hydrological connectivity tends to enhance dispersal-driven assembly, whereas environmental stability promotes niche-based selection [59].

Co-occurrence network analysis further revealed differences in microbial interaction patterns between the two zones. The bacterial network in the waterward zone contained more nodes and edges, indicating a more complex and highly connected microbial interaction structure (Figure 5). Increased connectivity among microbial taxa may enhance ecological interactions such as metabolic cooperation or resource sharing, potentially contributing to greater community resilience under hydrological disturbances [60,61,62]. In addition, network robustness analysis based on natural connectivity indicated that the microbial network in the waterward zone was more resistant to simulated disturbances than that in the landward zone. Natural connectivity is widely used as a topological indicator of network stability because it reflects the redundancy of alternative interaction pathways within a network [63]. Networks with higher natural connectivity generally possess more redundant connections, allowing them to maintain overall structural integrity even when some nodes are removed. Therefore, the higher natural connectivity observed in the waterward microbial network suggests a stronger capacity to buffer environmental disturbances.

The identification of keystone taxa further supports this interpretation. In the waterward zone, Methylotenera was identified as a module hub, whereas Nocardioides played a central role in the landward network. Keystone taxa are thought to disproportionately influence community structure and ecological processes relative to their abundance [64]. Their presence may therefore contribute to maintaining network connectivity and ecological stability under different hydrological conditions.

### 4.3. Multiple Factors Contributing to Functional Differences

Microbial community composition and interaction networks not only determine structural characteristics but may also influence the potential metabolic capabilities of microbial communities. In this study, functional potentials were inferred using the FAPROTAX database, revealing differences in predicted metabolic pathways related to carbon, nitrogen, and sulfur cycling across the aquatic–terrestrial ecotone (Figure 6).

Environmental conditions likely play an important role in shaping these functional patterns. In the waterward zone, higher soil moisture and elevated nutrient levels may create more reduced soil environments and greater substrate availability. Such conditions can favor microbial taxa associated with anaerobic or redox-sensitive processes, potentially increasing the relative importance of functions such as denitrification or methane-related metabolism [23,65,66]. By contrast, the landward zone experiences lower soil moisture and more stable oxygen availability, conditions that typically favor aerobic metabolic pathways and the decomposition of complex organic substrates [67]. These functional contrasts reflect well-established biogeochemical principles, whereby shifts in oxygen availability regulate the balance between oxidative and reductive pathways across soil systems [68]. These contrasting environmental conditions may therefore promote distinct functional potentials along the hydrological gradient. Different environmental variables may also influence microbial communities through distinct ecological mechanisms. Soil moisture can regulate oxygen diffusion and redox conditions within soil microhabitats, thereby influencing microbial metabolic pathways. In contrast, nutrient variables such as TP may primarily influence microbial activity by altering substrate availability or nutrient limitation [69,70].

Structural equation modeling further clarified the relationships among environmental variables, microbial community structure, and functional potential. The results suggested that environmental factors affected predicted microbial functions mainly through indirect pathways involving changes in community composition, α-diversity, and network stability (Figure 7). Among these factors, network stability exhibited the strongest positive association with predicted functional potential, implying that well-connected microbial interaction networks may support the maintenance of diverse metabolic capabilities. Ecological theory suggests that complex microbial networks often contain greater functional redundancy and cooperative interactions, which may enhance the ability of microbial communities to sustain ecosystem processes under environmental variability [71].

It is important to note that the functional profiles reported here were inferred from taxonomic information using the FAPROTAX database rather than directly measured through metagenomic or biochemical approaches. Therefore, these results should be interpreted as indicators of potential microbial functions rather than direct evidence of in situ metabolic activity. In addition, the relatively limited sample size, despite replicated sampling across multiple regions, may constrain the robustness of certain analyses, particularly those sensitive to sample size, such as null model inference, co-occurrence network construction, and structural equation modeling. Nevertheless, the overall consistency across different analytical approaches supports the robustness of the observed patterns. Future studies integrating larger sample sizes, metagenomic sequencing, and process-based measurements are needed to further validate these findings.

## 5. Conclusions

This study demonstrates that environmental heterogeneity driven by water-level fluctuations is a key factor regulating bacterial community structure, assembly processes, and functional potential in the aquatic–terrestrial ecotone of the Danjiangkou Reservoir. Waterlogging conditions, characterized by increased soil moisture and nutrient availability, promoted higher bacterial α-diversity, enhanced the relative importance of stochastic processes in community assembly, and supported more complex and stable microbial co-occurrence networks. In contrast, the relatively stable and oxic conditions in the landward zone strengthened deterministic selection and were associated with simpler network structures. Functional predictions further revealed clear metabolic differentiation along the hydrological gradient, with the waterward zone enriched in anaerobic processes such as nitrate reduction, denitrification, and methane metabolism, whereas the landward zone showed higher representation of aerobic functions including cellulose degradation and ammonia oxidation. Environmental factors indirectly regulated community stability and functional potential by shaping community composition and diversity. Overall, water-level fluctuations jointly regulate moisture and nutrient availability, thereby restructuring microbial community assembly, network stability, and functional potential across the ecotone. These findings highlight the central role of hydrological processes in governing microbially mediated ecosystem functions and provide a scientific basis for the management and restoration of reservoir riparian zones.

## Data Availability

The raw data supporting the conclusions of this article will be made available by the authors on request.

## Supplementary Materials

The following supporting information can be downloaded at: https://www.mdpi.com/article/10.3390/microorganisms14040923/s1, Table S1: Sampling sites in the Danjiangkou Reservoir; Table S2: Soil physicochemical factors in Waterward and Landward zones; Figure S1: Rarefaction curves of all samples in the Waterward and Landward zones; Figure S2: Comparison of Relative Abundance of Waterward and Landward Bacteria at phylum level; Figure S3: The bacterial community species composition of each site at family level; Figure S4: Quantifying the main environmental factors driving bacterial communities through Hierarchical partitioning analysis. (a) Waterward zones; (b) Landward zones; Figure S5: Niche Breadth of Bacterial Communities in the Waterward and Landward Zones; Figure S6: Robustness analysis is shown as the relationships between network natural connectivity and the proportion of removed nodes; Figure S7: Robustness analysis based on multiple edge and node removal strategies and robustness measurement; Figure S8: Heatmap showing the FAPROTAX functional prediction analysis of bacterial communities.
