# Supplementary material for: Characteristics, Assembly Processes and Stability of Bacterial Communities in Aquatic–Terrestrial Ecotone: A Case Study of Danjiangkou Reservoir, China
Source: Microorganisms. 2026 Apr 19;14(4):923. doi: 10.3390/microorganisms14040923 (PMC13118914; doi:10.3390/microorganisms14040923)
Supplement: Supplementary file 1 [file microorganisms-14-00923-s001.zip › microorganisms-4234735-supplementary.pdf]

Characteristics, Assembly process and Stability of Bacterial Communities in Aquatic–Terrestrial Ecotone: A Case Study of Danjiangkou Reservoir, China

Xucong Lyu [1], Junjun Mei [1], Haiyan Chen [2,3], Huatao Yuan [1,2,4,*], Jing Dong [1], Xiaofei Gao [1], Jingxiao Zhang [1], Yunni Gao [1,2,4] and Xuejun Li [1,2,4,*]

1 College of Fisheries, Henan Normal University, Xinxiang 453007, China; lvxvcong@outlook.com (X.L.); meijunjun2025@163.com (J.M.); happydj111@163.com (J.D.); xiaofeigao1989@163.com (X.G.); zhangjingxiao@htu.edu.cn (J.Z.); gaoyn@htu.cn (Y.G.)
2 Observation and Research Station on Water Ecosystem in Danjiangkou Reservoir of Henan Province, Nanyang 474450, China; haiyanch@126.com
3 Ecological Environment Monitoring and Emergency Center of the Source of South-to-North Water Diversion Project in Henan Province, Nanyang 474475, China
4 The National Ecological Quality Comprehensive Monitoring Station (Hebi Station), Hebi 458000, China
* Correspondence: yuanhuatao@htu.edu.cn (H.Y.), xjli@htu.cn (X.L.)

Tab. S1 Sampling sites in the Danjiangkou Reservoir.

Name	Sample sites	Longitude	Latitude
Danjiang	DJC1	111.569716	32.861461
	DJC2	111.568591	32.858188
	DJC3	111.567079	32.855832
	DJF1	111.588212	32.864832
	DJF2	111.588212	32.864832
	DJF3	111.579836	32.855406
Songgang	SGC1	111.623351	32.819912
	SGC2	111.631000	32.816403
	SGC3	111.637143	32.814165
	SGF1	111.639071	32.836440
	SGF2	111.644130	32.829982
	SGF3	111.644130	32.829982
Nangang	NGC1	111.650979	32.754485
	NGC2	111.651282	32.752295
	NGC3	111.654700	32.749592
	NGF1	111.659691	32.758608
	NGF2	111.662475	32.754694
	NGF3	111.663137	32.751945
Diaoshuikou	DSKC1	111.640343	32.716441
	DSKC2	111.639669	32.713806
	DSKC3	111.638448	32.709818
	DSKF1	111.652178	32.720571
	DSKF2	111.652093	32.716939
	DSKF3	111.648935	32.711100

Tab. S2 Soil physicochemical factors in Waterward and Landward zones.

	Waterward	Landward
NO_3 (mg/kg)	23.59±42.19a	5.29±5.81b
NH_3-N (mg/kg)	29.86±17.59a	9.93±0.55b
TP (g/kg)	0.94±0.27a	0.51±0.73b
TN (g/kg)	1.55±0.87a	1.07±0.74a
Ca (mg/kg)	23.20±17.12a	7.54±2.23b
Mg^{2+} (mg/kg)	3.87±2.65a	1.36±0.75b
Fe (mg/kg)	21.90±3.13b	29.69±3.63a
SOM (g/kg)	18.29±4.12a	16.44±4.3a
SMC (%)	15.27±5.21a	3.69±1.91b
pH	7.12±0.11a	7.25±0.23a

Note: Values are shown as the mean ± standard deviation (SD). Different letters indicate significant differences (Kruskal-Wallis test, $p < 0.05$)

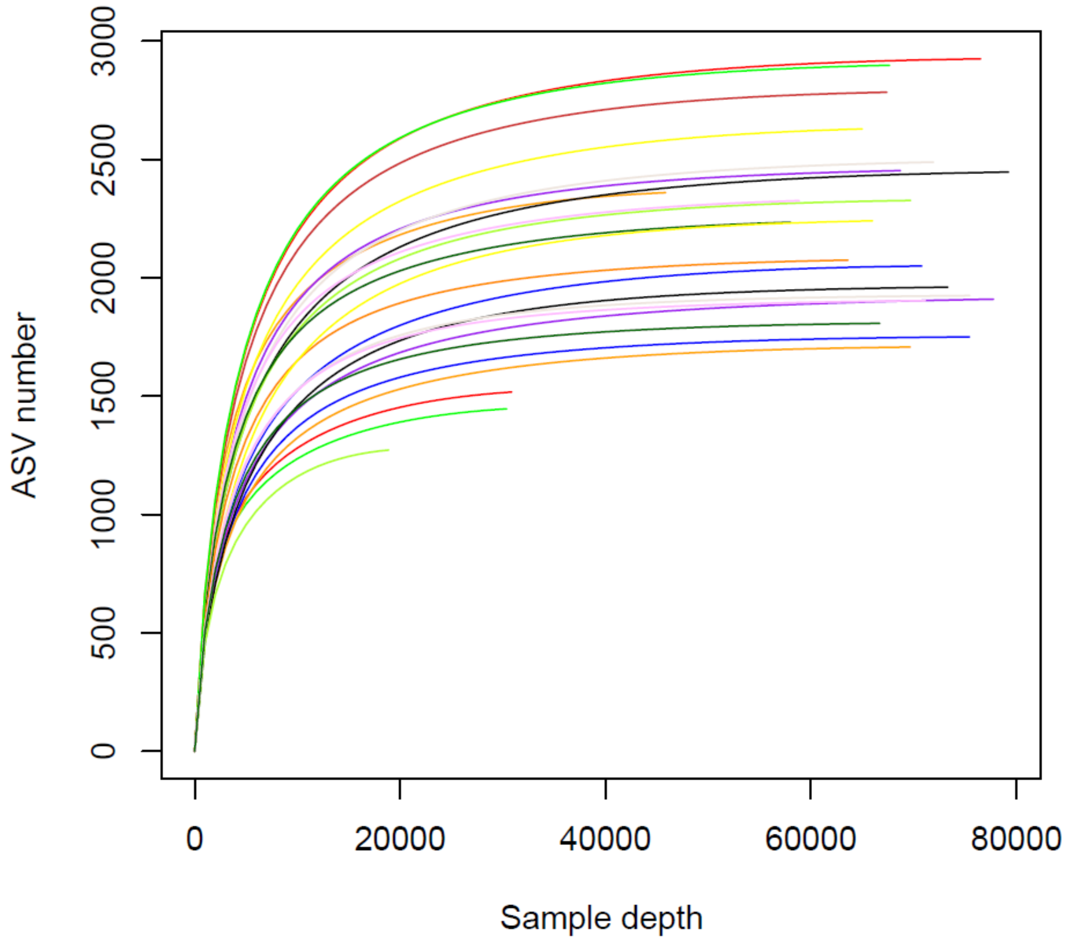

Fig. S1 Rarefaction curves of all samples in the Waterward and Landward zones.

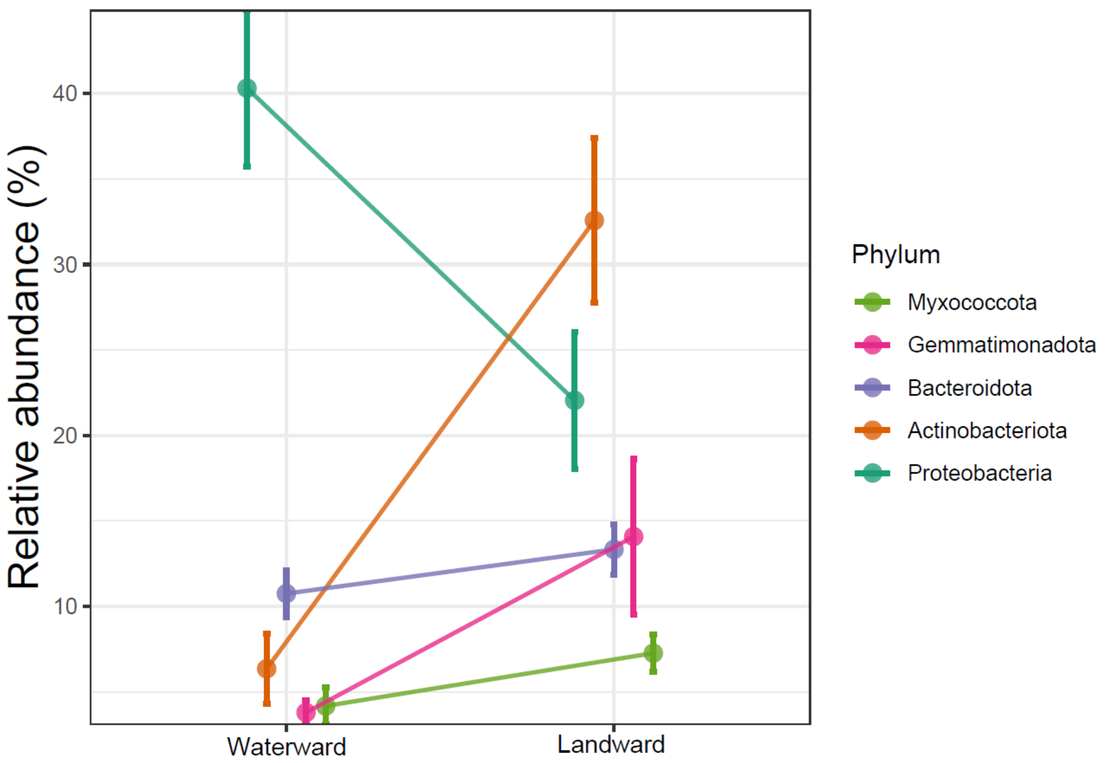

Fig. S2 Comparison of Relative Abundance of Waterward and Landward Bacteria at phylum level.

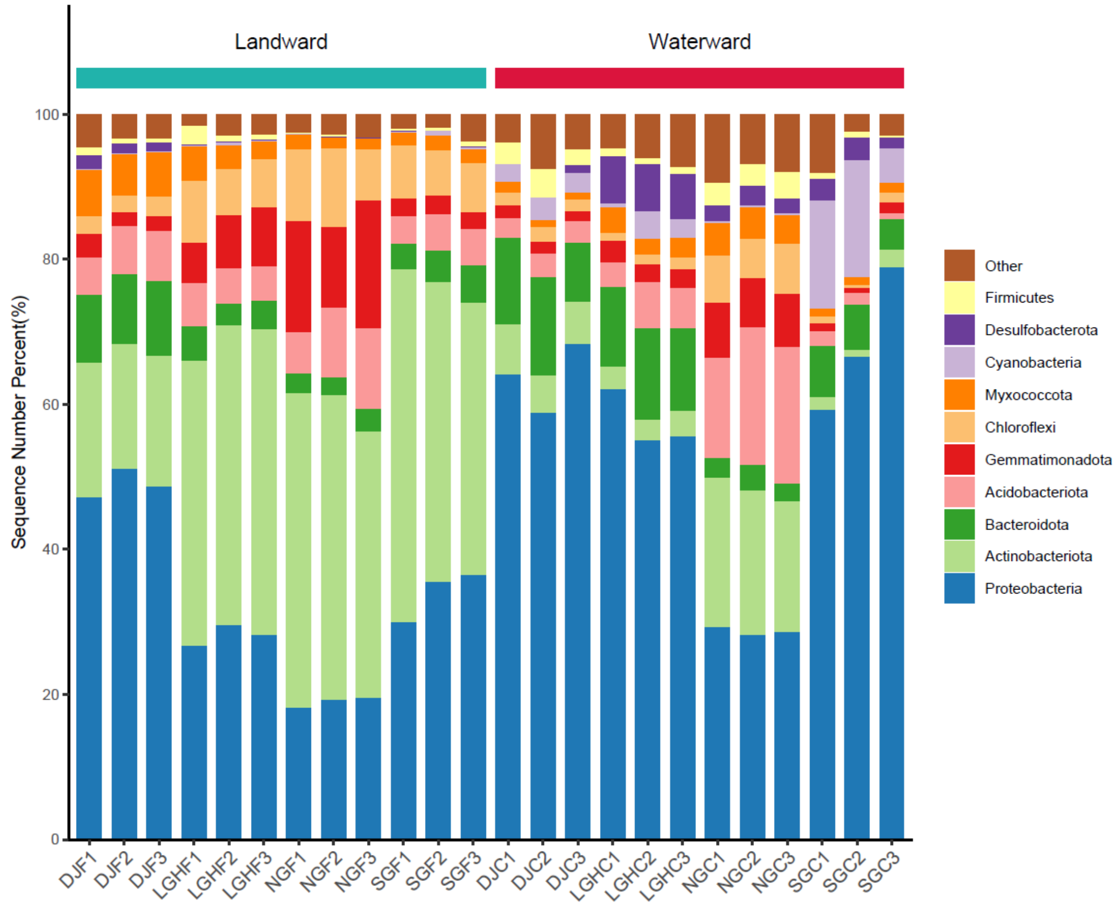

Fig. S3 The bacterial community species composition of each site at family level.

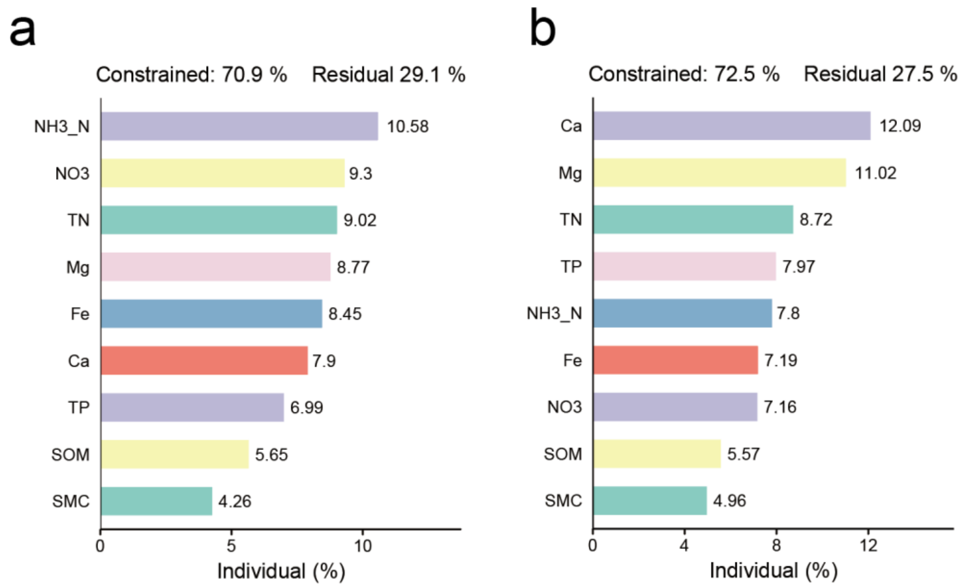

Fig. S4 Quantifying the main environmental factors driving bacterial communities through Hierarchical partitioning analysis. (a)Waterward zones; (b)Landward zones.

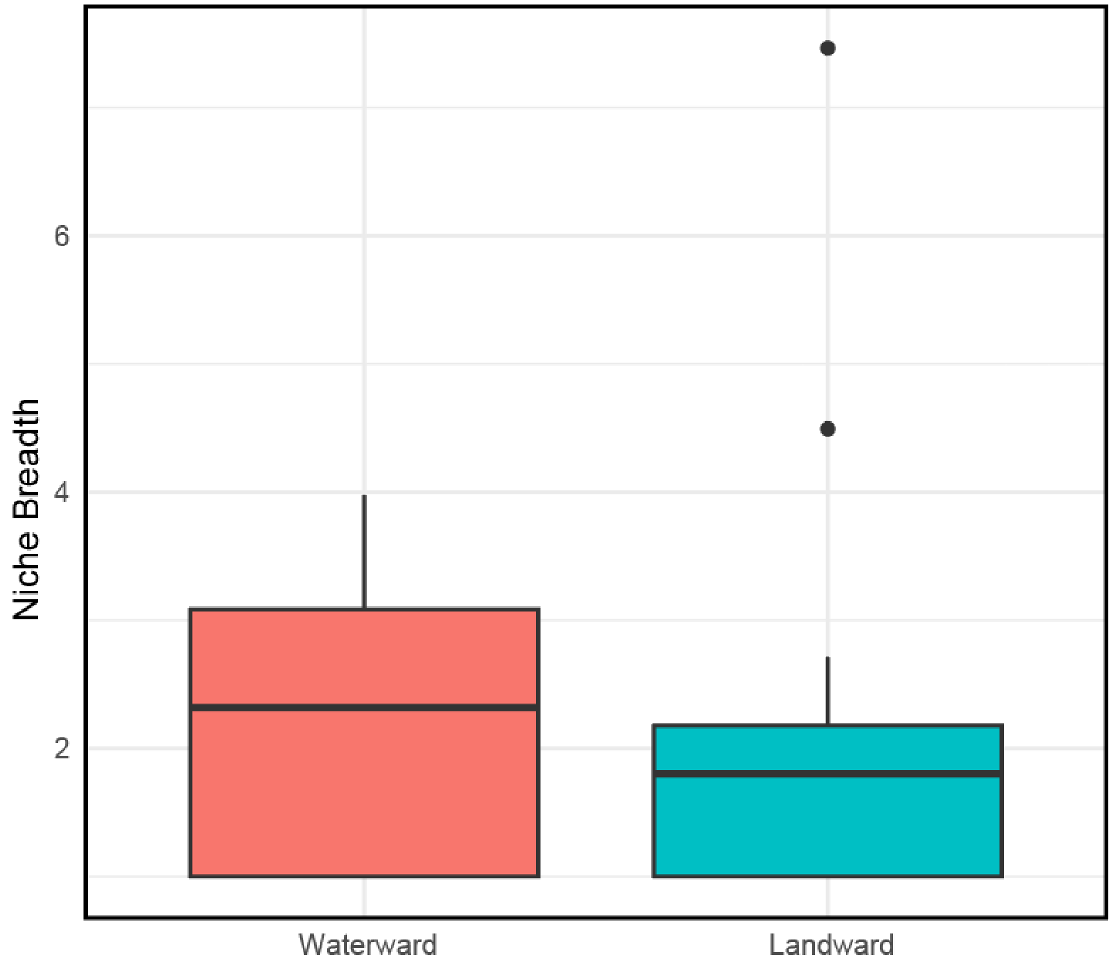

Fig. S5 Niche Breadth of Bacterial Communities in the Waterward and Landward Zones

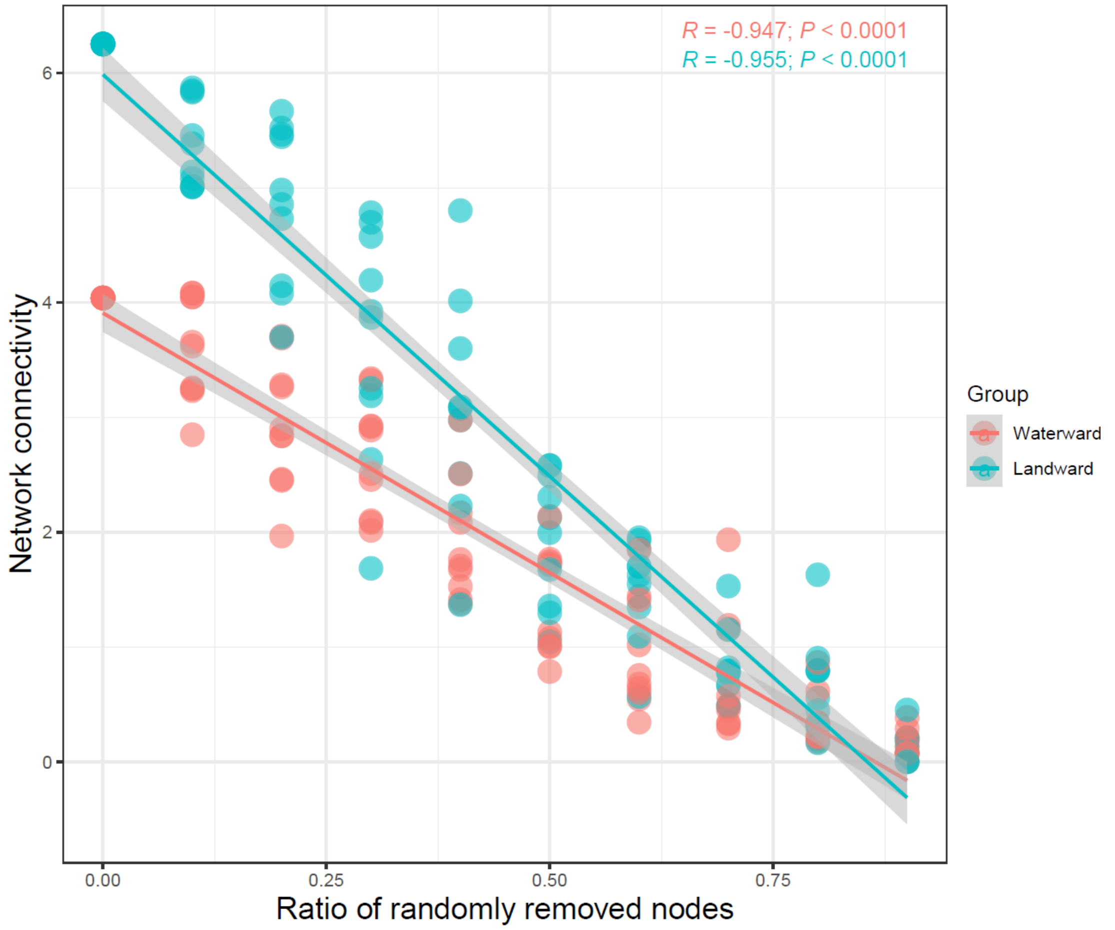

Fig. S6 Robustness analysis is shown as the relationships between network natural connectivity and the proportion of removed nodes.

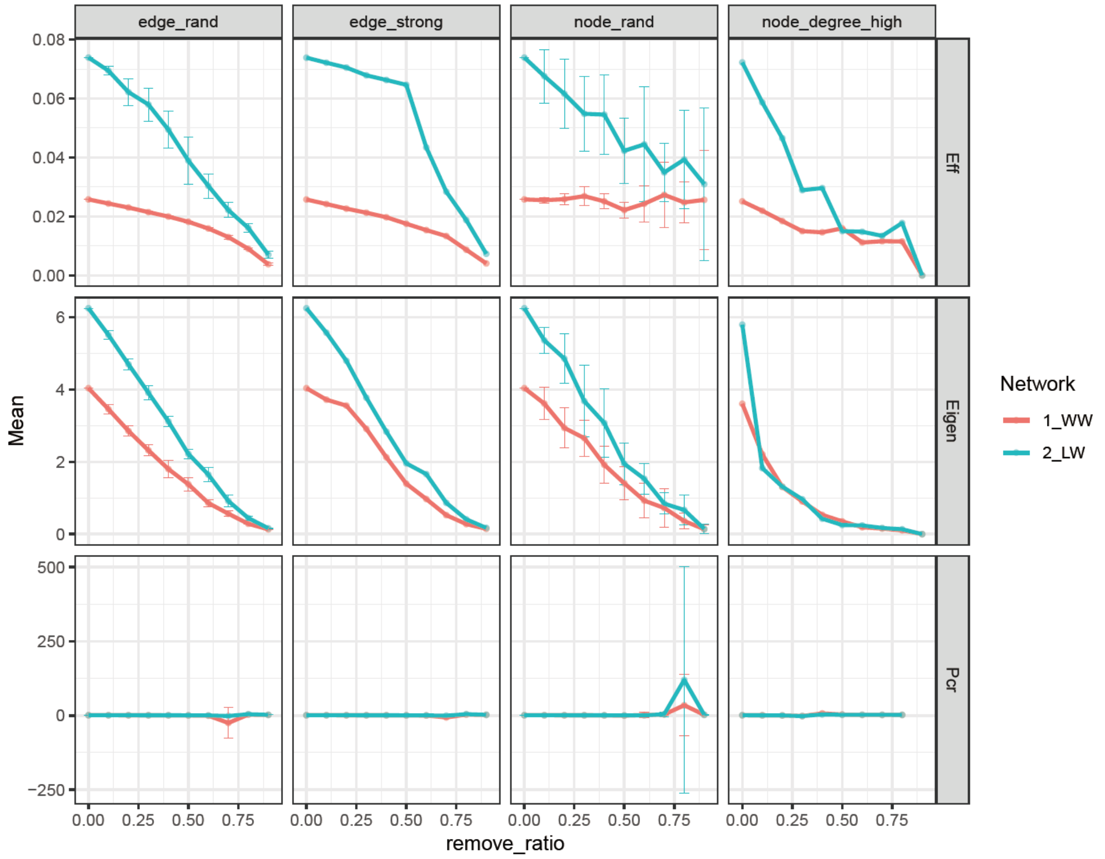

Fig. S7 Robustness analysis based on multiple edge and node removal strategies and robustness measurement

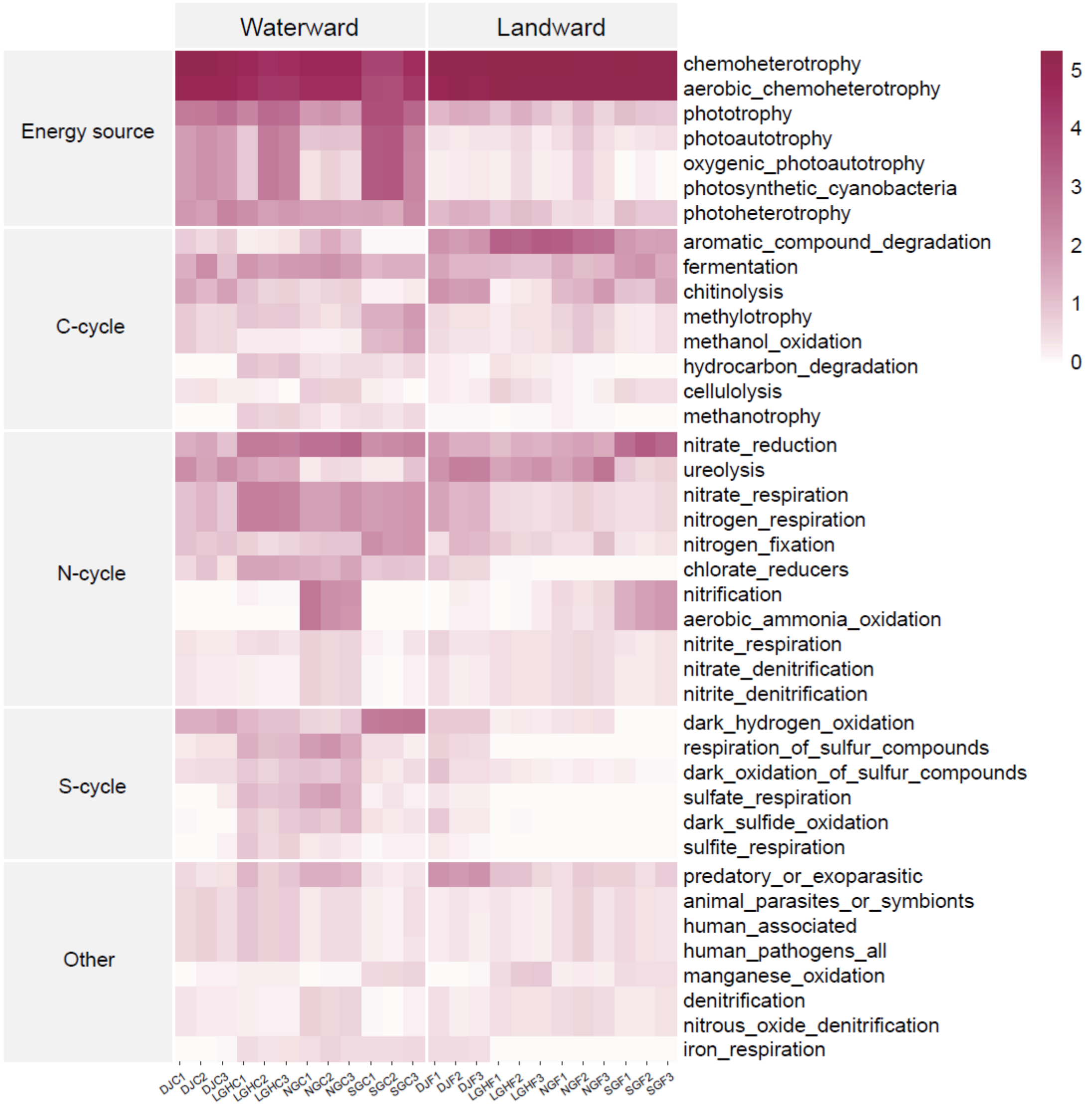

Fig. S8 Heatmap showing the FAPROTAX functional prediction analysis of bacterial communities
